# Large dose means significant effect – dose and effect relationship of Chi-Dan-Tui-Huang decoction on alpha-naphthylisothiocyanate-induced cholestatic hepatitis in rats

**DOI:** 10.1186/s12906-015-0637-0

**Published:** 2015-04-02

**Authors:** Yanling Zhao, Xiao Ma, Jiabo Wang, Ruiqing Wen, Lei Jia, Yun Zhu, Ruisheng Li, Ruilin Wang, Jianyu Li, Lifu Wang, Yonggang Li, Xiaohe Xiao

**Affiliations:** China Military Institute of Chinese Medicine, 302 Military Hospital of China,100#, the 4th Ring Road, Beijing, 100039 P R China; Pharmacy College, Chengdu University of Traditional Chinese Medicine, Chengdu, 611137 China; Beijing Haidian District Institute for Drug Control, Beijing, 100083 P R China; School of Chinese Medicine, The University of Hong Kong, Estates Building, 10 Sassoon Road, 999077, Hong Kong, 100039 P R China; Department of Integrative Medical Center, 302 Military Hospital of China, 100#, the 4th Ring Road, Beijing, 100039 P R China; Research and Technology Service Center, 302 Military Hospital of China, 100#, the 4th Ring Road, Beijing, 100039 P R China

**Keywords:** Chi-Dan-Tui-Huang decoction, *Radix Paeoniae* Rubra, Cholestatic hepatitis

## Abstract

**Background:**

Large dose application of traditional Chinese medicines has attracted more and more attentions in recent years. However, the scientific connotation of large dose application has not been clarified so far. The present study was designed to investigate the protective effects of Chi-Dan-Tui-Huang decoction (CDTHD) against Alpha-naphthylisothiocyanate (ANIT) induced acute cholestatic hepatitis in rats and explore the dose-effect relationship of CDTHD as a reference for clinical application.

**Methods:**

The administration of CDTHD at a series of doses was performed twice each day for 5 days. The acute cholestasic hepatitis models were induced by intragastric administration of ANIT on the third day of CDTHD administration. Then, the protective effects on cholestatic hepatitis were investigated by examining the following parameters: body weights of rats, morphological and histopathological liver changes, the levels of serum biomarkers including alanine aminotransferase, aspartate aminotransferase, alkaline phosphatase, total bilirubin, direct bilirubin and γ-glutamyltranspeptidase. Furthermore, the dose-effect relationship was investigated with the application of correspondence analysis.

**Result:**

In the tested range of doses, CDTHD at the maximum tolerance dose did not show any toxicity as time went on. The efficacy result showed that CDTHD from 21.6 g/kg⋅d to 86.4 g/kg⋅d exhibited significant hepatoprotective effect against ANIT-induced acute cholestatic hepatitis. It alleviated liver injury and reversed adverse biochemical and histopathological changes in a dose-dependent manner. Correspondence analysis showed that *Radix Paeoniae* Rubra in CDTHD was the main effective component and CDTHD could enhance the integrated efficacy in dose-dependent manner.

**Conclusions:**

CDTHD is beneficial to liver protection in a dose-dependent manner. Especially large dose demonstrates potent efficacy and *Radix Paeoniae* Rubra in the formula contributes the main effect on ANIT-induced acute cholestatic hepatitis without toxicity.

## Background

Complementary and alternative medicine (CAM), including Traditional Chinese Medicines (TCMs), has gained worldwide popularity over recent years [1,2]. The use of Chinese herbal medicine for the treatment of various disorders including liver diseases has attracted increasing attention [3,4]. In China, traditional Chinese herbs with modest side effects demonstrate potent efficacy to cholestasis [5-7]. Cholestasis, commonly induced by virus or iatrogenicity (drug-caused), is a clinical syndrome and presents as low bile flow from the liver to the duodenum [8]. Without proper treatment, cholestasis leads to jaundice and hypercholesterolemia, and later aggravated outcomes including cholestatic hepatitis, hepatic fibrosis, cirrhosis or even clinical sign of liver failure [9].

Chi-Dan-Tui-Huang decoction (CDTHD), serving as a famous decoction in cholestasis treatment, shows significant therapeutic effect in cholestasis [10,11]. It is composed of *Radix Paeoniae* Rubra, *Salvia miltiorrhiza* Bge, *Trichosanthis* Fructus and *Puerariae Lobatae* Radix. The doses of herbs in original formula are 150 g, 30 g, 30 g and 30 g respectively. Furthermore, in some cases, the dose of *Radix Paeoniae* Rubra reaches up to 200 g which is ten times more than the conventional dosage and shows remarkable effect with no toxicity and side effect [12,13]. In addition, *Radix Paeoniae* Rubra has been frequently used at large dose for treatment of acute cholestatic hepatitis and a number of clinical cases have demonstrated the affirmative effectiveness of CDTHD [14-16].

It is a scientific question in Traditional Chinese Medicines that why these herbs, such as *Radix Paeoniae* Rubra, can be used at a large dose. It is well known in western medicine that as the dose increases, toxicity and side effects accordingly rise. However, the administration of Chinese herbs at large doses usually shows good therapeutic effects with no or slight side effects [17]. Since ancient times, Chinese physicians usually paid particular attention to the doses of Chinese herbs because they believed that the dosage of Chinese herbs played a pivotal role in clinical situations and directly determined therapeutic efficacy. That is the basis for the popular old saying that the dose of TCMs should be kept as secret as possible and should not be shared. The use of Chinese herbs in large doses has been widely documented in TCM ancient records. For example, in *Yi Xue Zhong Zhong Can Xi Lu*, the dose of *Gypsum Fibrosum* reached up to 300 g per day in Bai-Hu-Decoction, which is thirty times the dose commonly used today (10 g per day) [18]. Furthermore, in *Li Ke Medical Records*, the dose of *Radix Aconiti Lateralis Preparata* in Po-Ge-Jiu-Xin-Decoction was set at 500 g per day, which is ten times compared with the regular dosage in Chinese Pharmacopoeia, and exhibited remarkable therapeutic effects [19]. The clinical evidence also verified the efficacy of CDTHD in treating acute cholestasis. However, the scientific connotation of dose-effect relationship of CDTHD for treating acute cholestatic hepatitis has not been clarified thus far.

**Figure 1:**
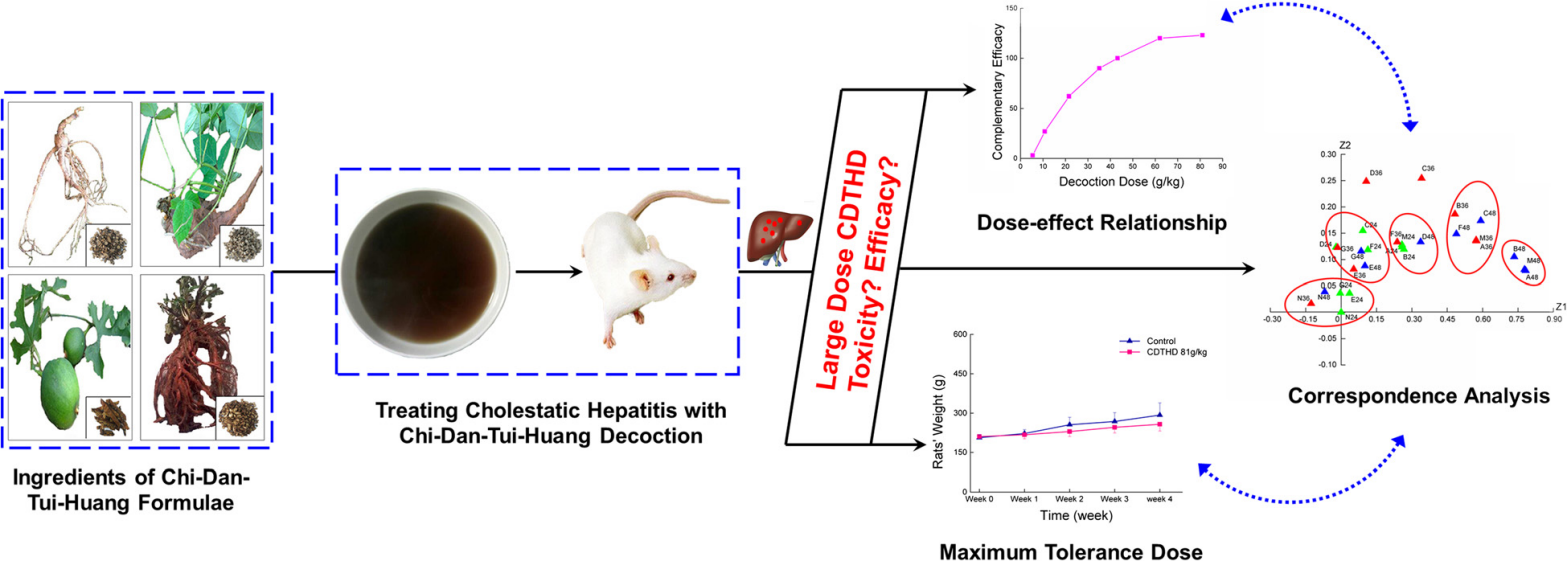
**Research strategy of CDTHD in large dose for cholestatic hepatitis.**

Alpha-naphthylisothiocyanate (ANIT), a compound for inducing hepatobiliary toxicity in vivo, is a common agent establishing animal models of cholestasis. The pathological and biochemical changes of ANIT-treated animals are similar to those of cholestatic hepatitis [20,21]. In present study, different doses of CDTHD were administered to rats with ANIT-induced acute cholestatic hepatitis. The following properties were investigated in this study: morphological and histopathological liver changes and serum biochemical indices, including alanine aminotransferase (ALT), aspartate aminotransferase (AST), total bilirubin (TBIL), direct bilirubin (DBIL), alkaline phosphatase (ALP), and γ-glutamyltranspeptidase (γ-GT) levels. Additionally, the protective effects of CDTHD with regards to the liver injury of rats were investigated, and the dose-effect relationship was evaluated with correspondence analysis (CA). Furthermore, the appropriate and scientific dose of CDTHD for cholestatic hepatitis treatment was explored. The data in this study provide scientific details for the proper application of CDTHD and give an example of exploring large dose application in TCMs (Figure 1).

## Methods

### Chemicals and reagents

*Radix Paeon*iae Rubra (Chi Shao in Chinese), *Salvia miltiorrhiza* Bge (Dan Shen in Chinese), *Trichosanthis* Fructus (Gua Lou in Chinese) and *Puerariae Lobatae* Radix (Ge Gen in Chinese) were purchased from Lvye Co., Ltd. (Beijing, China) and identified by Professor Xiaohe Xiao, a taxonomist at China Military Institute of Chinese Materia Medica. HPLC-grade acetonitrile and formic acid were purchased from Merck Company Inc. (Merck, Darmstadt, Germany). Ultrapure water was prepared by a Milli-Q50 SP Reagent Water System (Millipore Corporation, MA, USA) for the preparation of samples and buffer solutions. Standard compounds which were purchased from National Institute for the Control of Pharmaceutical and Biological Products (Beijing, China) were identified by Ultra Performance Liquid Chromatography (UPLC). ANIT was purchased from Sigma Chemical Co. (St. Louis, MO) and dissolved in olive oil to a final concentration of 2% before use. Kits for assaying the levels of alanine aminotransferase (ALT), aspartate aminotransferase (AST), total bilirubin (TBIL), direct bilirubin (DBIL), alkaline phosphatase (ALP) and γ-glutamyl transpeptidase (γ-GT) were purchased from Mindray bio-medical electronics Co., Ltd. (Shenzhen, China). All other reagents or drugs used were of analytical grade.

### Ethics statement

This study was conducted in strict accordance with the recommendations of the Guidelines for the Care and Use of Laboratory Animals of the Ministry of Science and Technology of China. The animal protocol was approved by the Committee on the Ethics of Animal Experiments of the 302 Military Hospital (Approval ID: 09–056).

### Animals

90 healthy male Wistar rats (200–220 g weight) were obtained from Vital River Lab Animal Technology Co., Ltd. (Beijing, China) (License No. SCXK 2007–004). These animals were kept at constant room temperature with a 12-h light/dark cycle and given free access to water and standard laboratory chow. Rats were acclimated to laboratory conditions for 1 week prior to the experiments.

### Water extract of CDTHD and phytochemical analysis

CDTHD extract was prepared by the prescription which included 150 g of *Radix Paeoniae* Rubra, 30 g of *Salvia Miltiorrhiza* Bge, 30 g of *Trichosanthis* Fructus and 30 g of *Puerariae Lobatae* Radix. The herbs were cut into small pieces, mixed and soaked in water (1/10 w/v) for 0.5 h, then boiled with reflux extraction for 1.5 h. The filtrate was collected and the residue was extracted again by reflux with the same volume of boiled water for another 1.5 h. The filtrates were combined and evaporated to dry under reduced pressure. The ratio of raw herbs to powders was 0.38. Waters Acquity UPLC System (Waters Corporation, USA) was used for phytochemical analysis of CDTHD. Chromatographic separation of the constituents in CDTHD was detected by ACQUITY UPLC BEH column (1.7 μm, 2.1 mm × 100 mm) heated to 35°C in the column compartment, with a gradient elution of solvent A (0.1% formic acid) and solvent B (acetonitrile) as follows: 0 min, B 15%; 10 min, B 35%; 15 min, B 50%; 20 min, B 95%, hold for 5 min. The detected ultraviolet wavelength was 250 nm. The injection volume of sample was 5 μL, and the flow rate was set at 0.3 ml/min. All mobile phase solvents were filtered with Whatman 0.22 μm nylon filter.

### Experimental procedure

The Wistar rats were randomly divided into 9 groups with 10 rats in each group: normal (control) group (N, n = 10), ANIT model group (M, n = 10), ANIT + A-dose of CDTHD (5.4 g/kg⋅d, A, n = 10) group, ANIT + B-dose of CDTHD (10.8 g/kg⋅d, B, n = 10), ANIT + C-dose of CDTHD (21.6 g/kg⋅d, C, n = 10), ANIT + D-dose of CDTHD (43.2 g/kg⋅d, D, n = 10), ANIT + E-dose of CDTHD (86.4 g/kg⋅d, E, n = 10), ANIT + F-dose of CDTHD (35.1 g/kg⋅d, F, n = 10) and ANIT + G-dose of CDTHD (62.1 g/kg⋅d, G, n = 10). The C-dose was the original working dose, while the E-dose was the highest feasible dose of CDTHD. F-dose and G-dose only increased the dose of *Radix Paeoniae* Rubra compared with C-dose (Table 1). In all cases, the dosage was converted to the equivalent rat dose of rats based on the human clinical dose [12]. The highest feasible dose was the maximal amount of extract that can be totally dissolved to yield the volume that a rat’s stomach can hold [22]. The administration of CDTHD at A-G doses (5.4, 10.8, 21.6, 43.2, 86.4, 35.1, 62.1 g/kg⋅d) was performed twice each day for 5 days. The acute cholestasis hepatitis models were induced by intragastric administration of a 2% ANIT solution (in olive oil, 1: 50, w/v, 60 mg/kg) on the third day of CDTHD administration. The ANIT model group was intragastrically treated with 60 mg/kg ANIT and without any treatment of CDTHD. The rats in normal group was just administered normal saline each day and intragastrical treatment with the vehicle (olive oil) alone. Blood samples were collected from the ophthalmic vein at 24 h, 36 h and 48 h after ANIT and CDTHD administration. At the end of the 48 h period, bile excretion experiments were performed to measure the bile flow. A portion of the liver tissue was preserved in 10% formalin for histopathological studies, while the rest of the portion was washed and kept in a freezer (−20°C) for further analysis.

**Table 1:** **Detail of group administration**

**Group name**	**Compositions and ratio of drug**	**Dose (g/kg)**
Normal (N)	Normal saline	Normalized
ANIT model (M)	Normal saline	Normalized
ANIT + A-dose (A)	Chi Shao:Dan Shen:Gua Lou: Ge Gen = 37.5:7.5:7.5:7.5	5.4
ANIT + B-dose (B)	Chi Shao:Dan Shen:Gua Lou: Ge Gen = 75:15:15:15	10.8
ANIT + C-dose (C)	Chi Shao:Dan Shen:Gua Lou: Ge Gen = 150:30:30:30	21.6
ANIT + D-dose (D)	Chi Shao:Dan Shen:Gua Lou: Ge Gen = 300:60:60:60	43.2
ANIT + E-dose (E)	Chi Shao:Dan Shen:Gua Lou: Ge Gen = 600:120: 120: 120	86.4 (MTD)
ANIT + F-dose (F)	Chi Shao:Dan Shen:Gua Lou: Ge Gen = 300:30:30:30	35.1
ANIT + G-dose (G)	Chi Shao:Dan Shen:Gua Lou: Ge Gen = 600:30:30:30	62.1

There were the same contents of Chi Shao between D and F group, E and G group in CDTHD. However, the difference between the groups above was the ratio of Dan Shen, Gua Lou and Ge Gen versus Chi Shao.

### Subacute toxicity protocol

Two groups of 20 rats each (half male and half female) were intragastrically administered (ig) with 86.4 g/kg aqueous extract of CDTHD (the maximum tolerance dose, MTD) and normal saline (as control group) twice a day for four weeks respectively. Body weight and mortality were recorded after administration of CDTHD, while blood specimens were collected from the ophthalmic vein every week. At the end of week 4, all animals were fasted overnight and sacrificed after blood samples were collected. Liver tissues were stored at −80°C until use.

### Histopathlogical observation

Liver tissue samples were fixed and preserved in 10% neutral buffered formalin for 24 h. All fixed liver tissues were embedded in paraffin, and cut into sections (approximately 4–5 μm thick) using a microtome. Sections were stained with hematoxylin-eosin (H&E). Histopathological determination of the extent liver injury was conducted with a light microscopy.

### Bile excretion experiments

The rats were anaesthetized with urethane (1 g/kg, i.p.) and the bile duct was cannulated with PE10 polyethylene tube (inner diameter, 0.28 mm; outer diameter, 0.61 mm; Becton, Dickinson and Co., Franklin Lakes, NJ, USA). After the operation, the bile flow rate within 6 hours was recorded.

### Biochemical assay

Blood samples were collected from the ophthalmic vein for the assessment of the serum levels of ALT, AST, TBIL, DBIL, ALP and γ-GT. Blood serum was separated by centrifugation at 3000 × *g* for 10 min at 4°C. Then, serum was analyzed using a commercially available clinical test kit with a Mindray clinical analyzer BS 300 (Mindray bio-medical electronics Co., Ltd. China).

### Statistic analysis and correspondence analysis on biochemical indices

The data were expressed as the mean ± S.E.M. and analyzed with the SPSS software package, version 12.0. The differences between the group means were analyzed by one-way analysis of variance (ANOVA). The differences were considered to be statistically significant when P ≤ 0.05 and highly significant when P ≤ 0.01.

CA was performed to search for the integrated effect of formula at respective dose. The data were expressed as different time and administration with mean value of corresponding ALT, AST, TBIL, DBIL, ALP and γ-GT. The CA procedure was run by SAS 9.2. The integrated effect was measured by Euclidean distance among normal group, model group and other administration groups.

## Results

### Phytochemical analysis of CDTHD

**Figure 2:**
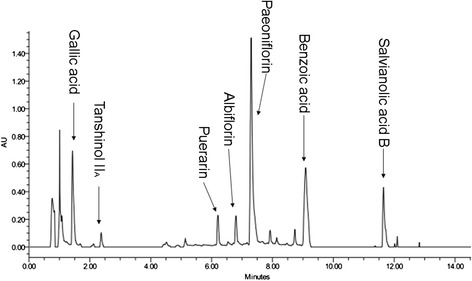
**UPLC fingerprint and composition of CDTHD.**

The typical UPLC fingerprint of water extract of CDTHD was shown in Figure 2. Most of the compounds were separated within 14 min according to the chromatograph. Seven compounds including Gallic acid, Tanshinol II_A_, Puerarin, Albiflorin, Paeoniflorin, Benzoic acid and Salvianolic acid B were detected and identified by comparing the t_R_ value of the corresponding reference substances. Furthermore, these compounds reflected most of the chemical information of CDTHD. Gallic acid and Benzoic acid widely existed in a variety of herbs such as Dan Shen, Chi Shao. Tanhinol II_A_ and Salvianolic acid B were the chemical representatives of Dan Shen. Paeoniflorin and Albiflorin were two of the major constituents derived from Chi Shao. As for Puerarin, it was the primary efficient composition of Ge Gen.

### Subacute toxicity study

**Figure 3:**
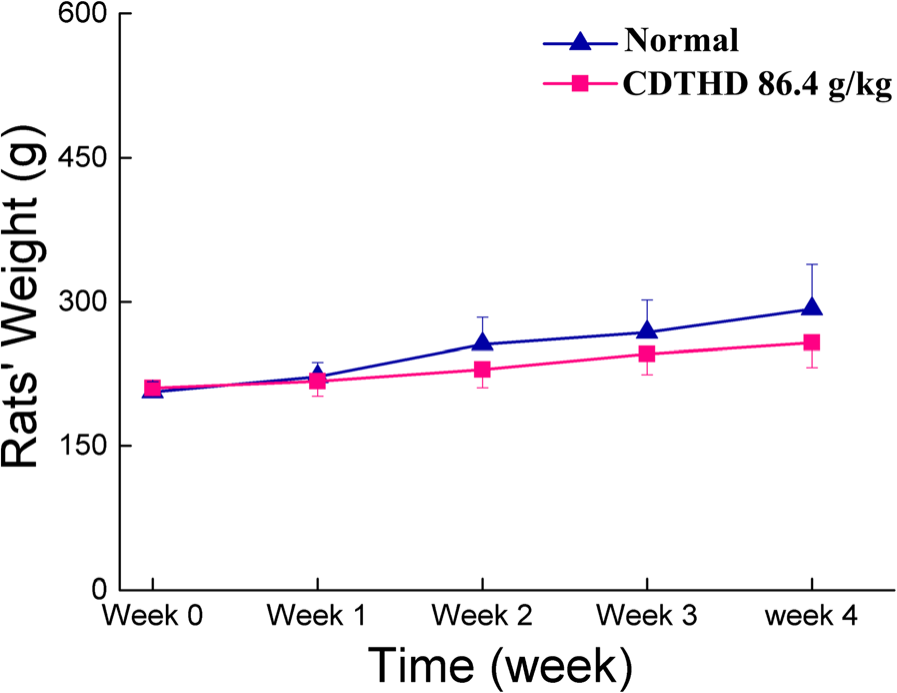
**Comparison of rats’ body weight between normal and CDTHD 86.4 g/kg groups.**

No dead rats were observed during our evaluation of the subacute toxicity of CDTHD. In addition, there were no obvious symptoms of subacute toxicity during the experiment. Our subacute toxicity study showed that treatment with 86.4 g/kg CDTHD caused a slight and time-dependent decrease in body weight. However, there was no significance in rats’ body weight between two groups (*P* > 0.05). Serum levels of multiple biomarkers including ALT, AST, TBIL, DBIL, ALP, γ-GT, BUN, CREA, GLU, ALB, TP and TCHO were analyzed. Data within four weeks were shown in Table 2 and 3. Compared with the normal group, there were almost no changes in CDTHD 86.4 g/kg treatment group in four weeks at any time points (*P* > 0.05). These results demonstrated that large dose of CDTHD which reached the maximum tolerance dose showed no obvious toxic effect in rats (Figure 3, Table 2, Table 3).

**Table 2:** **Serum ALT, AST, ALP, γ-GT, TBIL and DBIL levels in control and CDTHD 86.4 g/kg groups within 4 weeks**

**Time**	**Group**	**ALT(U/L)**	**AST(U/L)**	**ALP(U/L)**	**γ-GT(U/L)**	**TBIL(μmol/L)**	**DBIL(μmol/L)**
Week	Control	76.83 ± 9.24	236.24 ± 28.96	364.58 ± 38.64	13.65 ± 3.54	22.39 ± 0.83	13.68 ± 1.24
1	86.4 g/kg	70.52 ± 10.12	226.54 ± 22.53	357.26 ± 29.87	12.87 ± 2.49	23.68 ± 0.92	12.56 ± 0.66
Week	Control	72.54 ± 8.56	234.58 ± 30.16	361.87 ± 26.52	12.94 ± 3.64	21.36 ± 1.32	12.68 ± 0.87
2	86.4 g/kg	69.87 ± 7.31	223.65 ± 25.45	359.72 ± 33.62	11.56 ± 2.87	22.16 ± 0.93	11.63 ± 0.98
Week	Control	74.69 ± 8.78	232.75 ± 29.48	359.62 ± 25.53	13.15 ± 1.13	20.65 ± 1.13	12.11 ± 1.12
3	86.4 g/kg	68.23 ± 10.32	228.68 ± 23.54	354.35 ± 28.83	10.82 ± 2.62	22.61 ± 1.24	12.46 ± 0.88
Week	Control	72.15 ± 6.38	234.26 ± 28.75	359.26 ± 30.56	11.96 ± 3.64	21.51 ± 1.36	12.35 ± 1.18
4	86.4 g/kg	68.46 ± 7.52	227.45 ± 26.64	352.69 ± 33.69	11.84 ± 2.82	23.15 ± 1.24	13.29 ± 1.02

Data were shown as mean ± SE of 10 rats. There was no difference between two groups (*P* > 0.05).

**Table 3:** **Serum BUN, CREA, GLU, ALB, TP and TCHO levels in control and CDTHD 86.4 g/kg groups within 4 weeks**

**Time**	**Group**	**BUN(mmol/L)**	**CREA(μmol/L)**	**GLU(mmol/L)**	**ALB(g/L)**	**TP(g/L)**	**TCHO(mmol/L)**
Week	Control	7.62 ± 0.66	33.86 ± 3.91	5.82 ± 0.88	46.52 ± 2.33	74.65 ± 3.62	1.56 ± 0.18
1	86.4 g/kg	7.64 ± 0.53	34.62 ± 3.73	5.68 ± 0.75	45.46 ± 1.95	75.83 ± 4.24	1.53 ± 0.17
Week	Control	7.54 ± 0.45	32.24 ± 4.45	5.73 ± 0.64	45.23 ± 2.47	73.96 ± 3.37	1.52 ± 0.14
2	86.4 g/kg	7.61 ± 0.51	33.71 ± 3.64	5.77 ± 0.58	46.73 ± 2.14	74.83 ± 4.13	1.55 ± 0.16
Week	Control	7.51 ± 0.52	30.93 ± 4.26	5.69 ± 0.81	44.35 ± 2.62	73.91 ± 3.9	1.54 ± 0.15
3	86.4 g/kg	7.56 ± 0.61	33.95 ± 3.57	5.71 ± 0.72	45.87 ± 1.92	72.35 ± 3.62	1.56 ± 0.18
Week	Control	7.59 ± 0.48	32.66 ± 3.82	5.74 ± 0.69	43.63 ± 2.26	74.74 ± 4.36	1.51 ± 0.16
4	86.4 g/kg	7.55 ± 0.54	32.85 ± 4.37	5.76 ± 0.65	45.77 ± 2.74	73.25 ± 3.68	1.57 ± 0.15

Data were shown as mean ± SE of 10 rats. There was no difference between two groups (*P* > 0.05).

### Effect of CDTHD on histopathological changes

**Figure 4:**
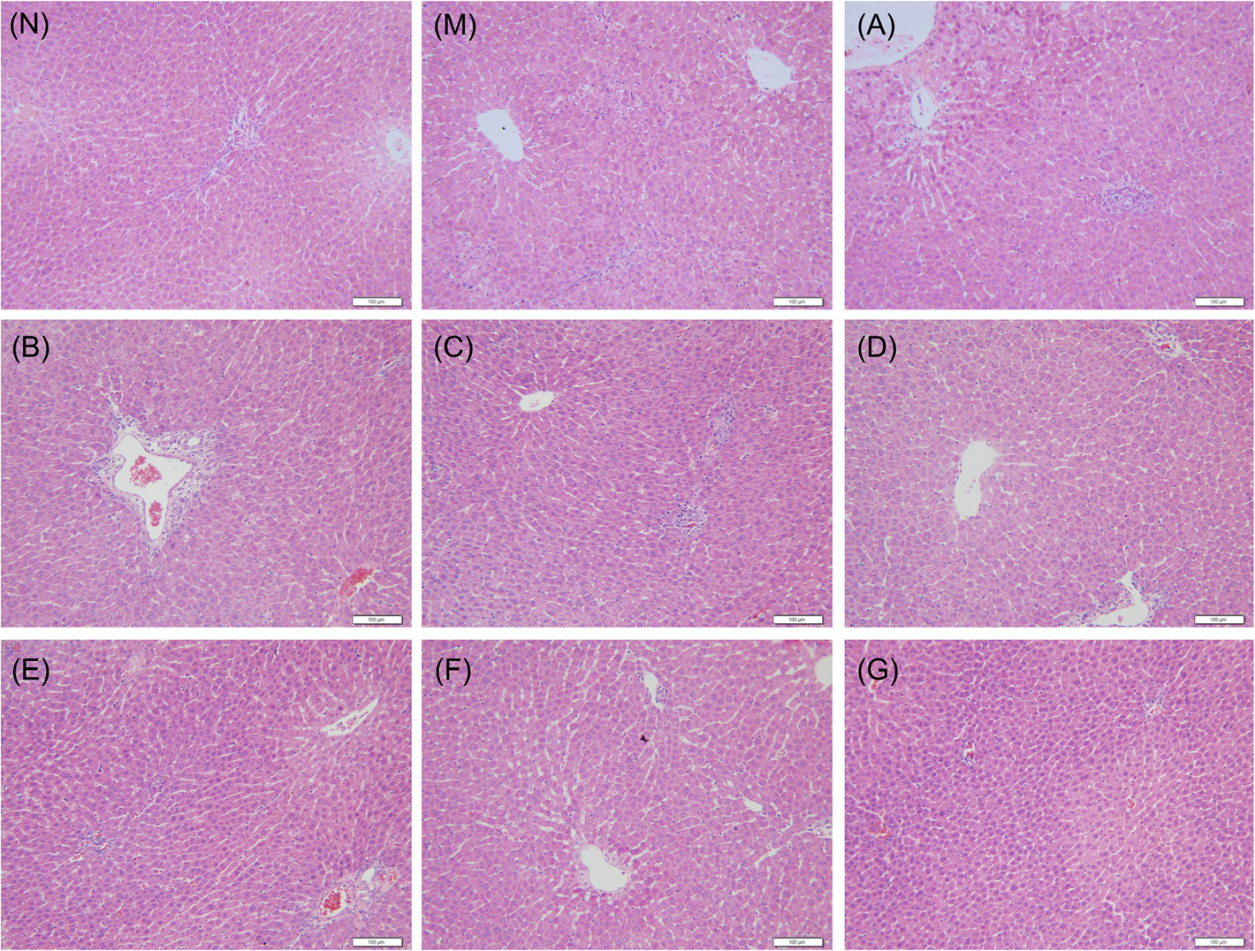
**Histopathological changes of groups treated with or without CDTHD in liver tissue. A**: 5.4 g/kg CDTHD, **B**: 10.8 g/kg CDTHD, **C**: 21.6 g/kg CDTHD, **D**: 43.2 g/kg CDTHD, **E**: 86.4 g/kg CDTHD, **F**: 35.1 g/kg CDTHD, **G**: 62.1 g/kg CDTHD.

As shown in Table 4 and Figure 4, the hepatic tissues of rats belonging to the normal (N) group exhibited no abnormal appearance or histological changes (Figure 4N). In the ANIT-induced model group (M), pathological changes of severe inflammatory cell infiltration, hepatocyte necrosis, hydropic degeneration, severe demolishment or loss of the interlobular ducts and cholestasis were observed in rat livers at 48 h (Figure 4M). Compared with M group, histopathologcial changes induced by ANIT were remarkably improved by higher doses of CDTHD in a dose-dependent manner and the histopathological changes were quite obvious, with almost complete recovery in C, D, E, F, G groups (Figure 4C, D, E, F and G), whereas treatment with lower doses (A and B) of CDTHD showed no obvious changes (Figure 4A and B).

**Table 4:** **Effect of CDTHD on histopathological changes in rat liver obtained at 48 h after ANIT administration (n = 10)**

**Group**	**Hepatocyte hydropic degeneration**	**Hepatocellular necrosis**	**Sinusoid enlargement**	**Inflammatory cells infiltration**
N	-	-	-	-
M	++	+++	+	++
A	++	+	+	+
B	++	+	-	-
C	-	+	-	-
D	-	-	-	-
E	-	-	-	-
F	-	-	-	+
G	-	-	-	-

Data were expressed as the mean of ten specimens for each group. + Mild, ++ Moderate, +++ Marked, − Negative. N: Normal, M: Model, A: 5.4 g/kg CDTHD, B: 10.8 g/kg CDTHD, C: 21.6 g/kg CDTHD, D: 43.2 g/kg CDTHD, E: 86.4 g/kg CDTHD, F: 35.1 g/kg CDTHD, G: 62.1 g/kg CDTHD.

### Effect of CDTHD on bile excretion

**Figure 5:**
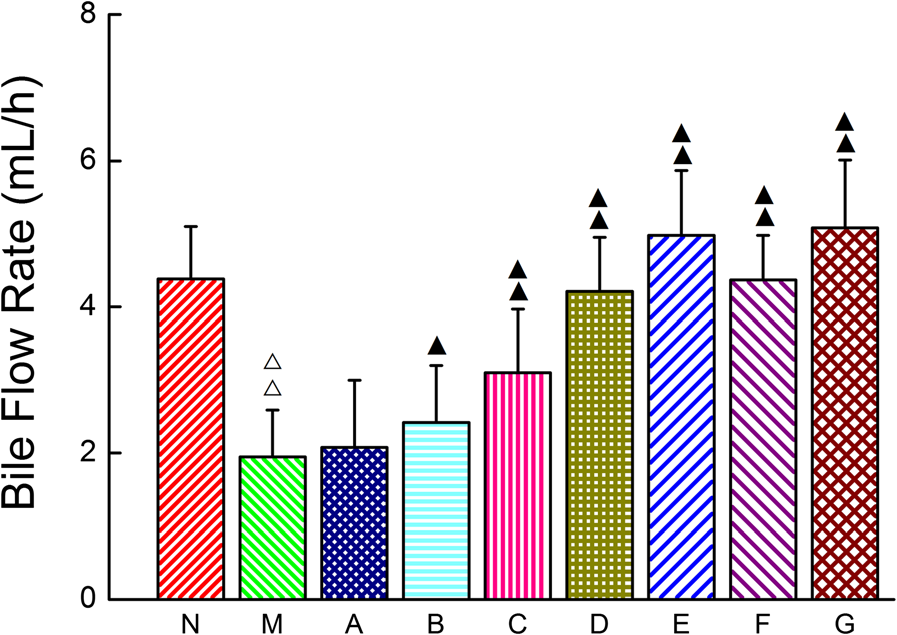
**Bile flow rate of CDTHD in different doses.** N: Normal, M: Model, A: 5.4 g/kg CDTHD, B: 10.8 g/kg CDTHD, C: 21.6 g/kg CDTHD, D: 43.2 g/kg CDTHD, E: 86.4 g/kg CDTHD, F: 35.1 g/kg CDTHD, G: 62.1 g/kg CDTHD. ^△^ *p* < 0.05, ^△△^ *p* < 0.01 compared with normal group. ^▲^ *p* < 0.05, ^▲▲^ *p* < 0.01 compared with model group.

As shown in Figure 5, the bile flow rate within 6 h was markedly suppressed in M group (1.71 ± 0.54 mL/h) compared with N group (4.75 ± 0.38 mL/h) (*P* < 0.01). Moreover, the bile flow rate was significantly increased from B to G group treated with CDTHD (*P* < 0.01, *P* < 0.05). Furthermore, the bile flow rate of D, E, F, G groups was almost the same as N group. In addition,F and G groups demonstrated similar bile flow rate compared with D and E groups respectively.

### Effect of CDTHD on serum biomarkers in rats with ANIT induced cholestasis

**Figure 6:**
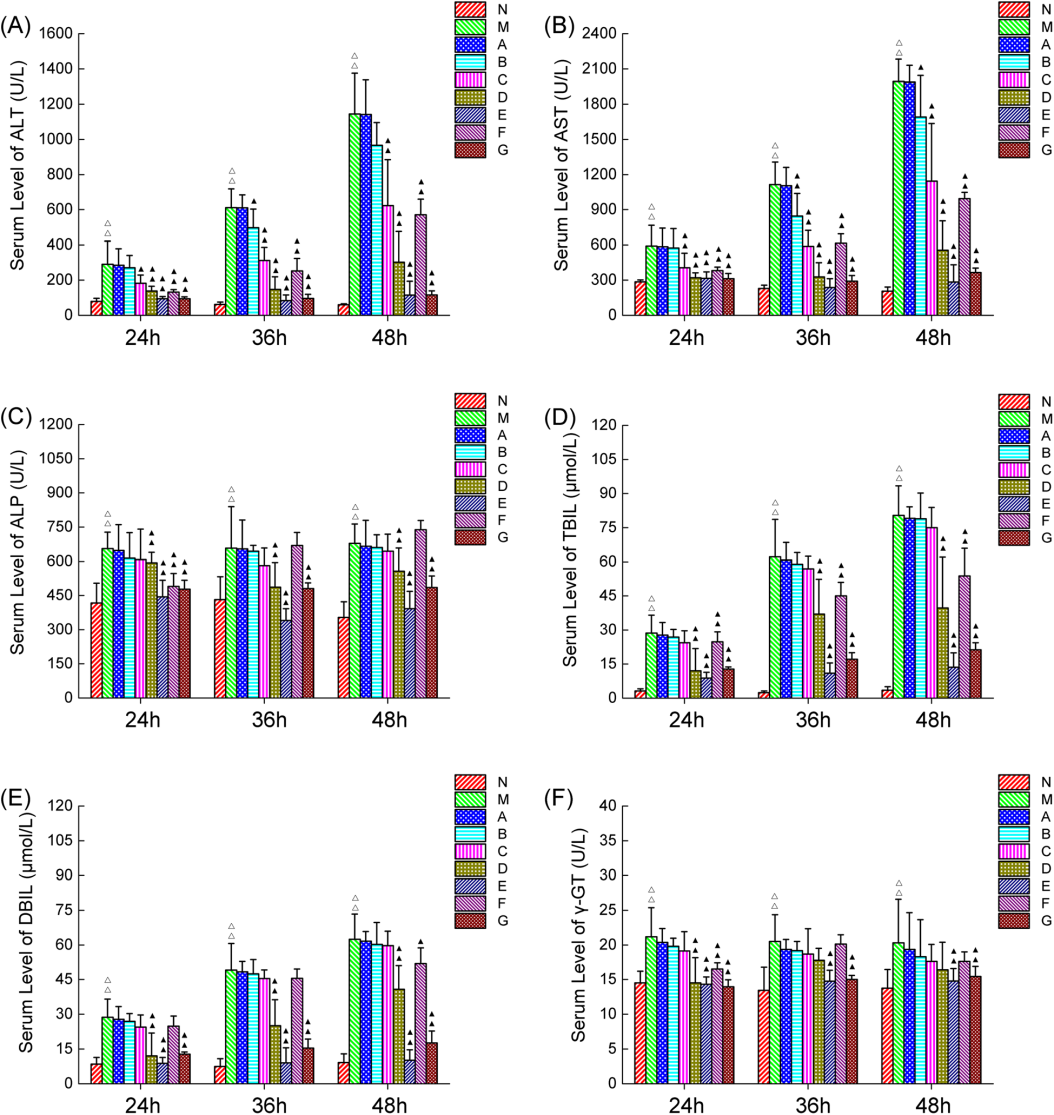
**Serum ALT, ALP, AST, TBIL, DBIL and γ-GT levels in 9 groups of rats treated with or without CDTHD.** N: Normal, M: Model, **A**: 5.4 g/kg CDTHD, **B**: 10.8 g/kg CDTHD, **C**: 21.6 g/kg CDTHD, **D**: 43.2 g/kg CDTHD, **E**: 86.4 g/kg CDTHD, **F**: 35.1 g/kg CDTHD, **G**: 62.1 g/kg CDTHD. ^△^ *p* < 0.05, ^△△^ *p* < 0.01 compared with normal group. ^▲^ *p* < 0.05, ^▲▲^ *p* < 0.01 compared with model group.

The levels of various serum biomarkers (ALT, AST, TBIL, DBIL, ALP and γ-GT levels) in each group at different time points were shown in Figure 6. Serum ALT, AST, TBIL, DBIL, ALP and γ-GT increased significantly in model group compared with normal group from 24 h to 48 h (*P* < 0.01) which were consistent with former records [21]. Moreover, the results showed that these biochemical indices such as ALT, AST TBIL and DBIL increased gradually within 48 h in model group. However, ALP and γ-GT kept almost the same among 24 h, 36 h and 48 h. Serum levels of ALT, AST, TBIL, DBIL, ALP and γ-GT decreased with CDTHD treatment in a dose-dependent manner compared with model group. In detail, A-dose (5.4 g/kg⋅d), B-dose (10.8 g/kg⋅d) and C-dose (21.6 g/kg⋅d) demonstrated no obvious improvement in ALP, TBIL, DBIL and γ-GT (*P* >0.05). However, high doses of CDTHD, such as D, E, F and G-dose, exhibited significant therapeutic effect in the treatment of acute cholestaic hepatitis in all serum indices. Furthermore, F and G groups demonstrated similar efficacy compared with D and E groups respectively. The data from the experiment suggested that large dose CDTHD was effective against cholestaic hepatisis.

### Correspondence analysis

**Figure 7:**
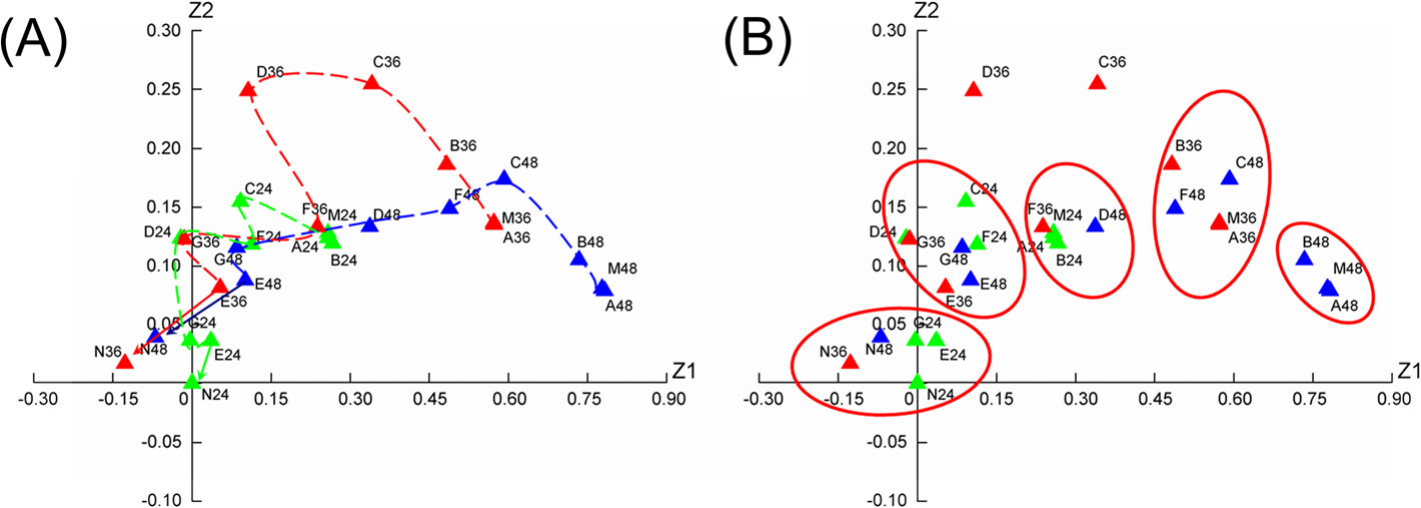
**Correspondence analysis on integrated efficacy and similarity of different doses CDTHD. A**: Correspondence analysis of time-dose-efficacy relationship, **B**: Effect clusters of similar groups. N24-G24: N group at 24 h to G group at 24 h, N36-G36: N group at 36 h to G group at 36 h, N48-G48: N group at 48 h to G group at 48 h.

Correspondence analysis provided a visualized impress of diverse data relationship. It was used to analyze the integrated efficacy and similarity of different doses of CDTHD in this research. As shown in Figure 7, the integrated efficacy was composed of serum biomarkers such as ALT, AST, TBIL, DBIL, ALP and γ-GT. The point depicted as rectangle in coordinate system with *Z1* (horizontal axis) and *Z2* (longitudinal axis) represented each investigated group. The name of each group in coordinate system expressed as N24 was the normal group at the end of 24 h. The Euclidean distance was to measure the relationship between two groups and was listed in Table 5.

**Table 5:** **Euclidean distance of different doses CDTHD**

**Group to group**	**24 h**	**36 h**	**48 h**
M to N (N to M)	0.0829531	0.5042984	0.719462
A to N	0.0817174	0.5047401	0.728083
B to N	0.0851343	0.4010343	0.6504139
C to N	0.0323821	0.2762745	0.4558273
D to N	0.015723	0.1084588	0.1745785
E to N	0.0025864	0.0368022	0.0314098
F to N	0.0268291	0.1468612	0.3239829
G to N	0.0013232	0.0237961	0.0298026
A to M	0.0000178	0.0000031	0.0000308
B to M	0.0001493	0.0104849	0.0024807
C to M	0.028301	0.00672042	0.0430982
D to M	0.0780845	0.2296862	0.1965076
E to M	0.0578047	0.2727777	0.4580789
F to M	0.0210222	0.1120853	0.0879489
G to M	0.0769024	0.3455155	0.4808165

As shown in Figure 7A and Table 5, the Euclidean distance from M to N gradually increased from 24 h (0.0829531) to 36 h (0.5042984) and to 48 h (0.719462), demonstrating that ANIT successfully induced cholestatic hepatitis and this injury would be aggravated with time going on. Secondly, the Euclidean distance from treating groups to normal group in 24 h, 36 h and 48 h displayed as M-N ≈ A-N ≈ B-N > C-N > D-N ≈ F-N > E-N ≈ G-N. On the contrary, the Euclidean distance from groups to model group in 24 h, 36 h and 48 demonstrated opposite result (N-M > E-M ≈ G-M > D-M ≈ F-M > C-M > B-M > A-M) compared with normal group. It indicated that the M, A, B groups were similar. Meanwhile, there was a close relationship between D and F as well as E and G. The D, E, F and G groups were close to normal group. Correspondence analysis also showed a direct relationship among different samples. As shown in Figure 7B, there were 5 main clusters gathering in the coordinate system. The first cluster containing N24, E24, G24, N36 and N48 was gathered by the short distance. Rats administered with ANIT recovered from the treatment of E-dose and G-dose of CDTHD at the initial 24 h with similar physical condition to the N group. The second cluster contained D24, F24, C24, E36, G36, E48 and G48. From this cluster, the efficacy of E-dose and G-dose at 36 h and 48 h contributed the same to D, F and C dose at 24 h. These doses, though at different time, demonstrated similar efficacy. The third cluster (A24, B24, M24, F36 and D48), the fourth cluster (B36, A36, M36, F48 and C48) and the fifth cluster (B48, M48 and A48) demonstrated slight effect or even no effect on cholestatic hepatitis. In addition, the injury degree deteriorated in these last three clusters as time went on.

## Discussion

It is believed that the mild dose application for treating several specific diseases always gets unsatisfactory efficacy in TCM. Meanwhile, under the particular condition, large dose application demonstrates potent effect [23]. However, the rationality of large dose is still being doubted, and it is unclear whether the toxicity or effect exists. In recent years, increasing attention has been paid to the relationship between dosage and effect [24,25]. Based on clinical evidences, large dose application has been further investigated in laboratory settings [26]. Previous investigations of the relationship between the dose and the efficacy/toxicity of Rheum officinale showed that the effects of Rheum officinale on normal animals and CCl4-treated animals respectively were entirely different. The conclusion was drawn from the experiments indicated that a drug would show its therapeutic effect when it was prescribed to patients with the correct indications, while it might produce deleterious effects in individuals as result of incorrect indications [27]. Moreover, it also implied that the dose of the drug is critical to its rational application in the clinic to balance the benefits and risks.

Cholestasis, which is characterized by the impairment of bile flow, often occurs in hepatocyte and intrahepatic biliary ductules, or sometimes as a result of extrahepatic obstruction of the bile ducts [28]. Interruption of bile flow leads to the accumulation of bile acids and other bile components in the liver and ultimately results in hepatobiliary toxicity [29]. ANIT is a hepatotoxican widely used in rodents to imitate human intrahepatic cholestasis over the years [30]. Furthermore, ANIT induced hepatotoxicity animal model is considered to be useful for evaluating the therapeutic effect of medicines [31,32].In this research, the relationship between the dose of CDTHD and protective effects was investigated under ANIT induced cholestatic hepatitis. We firstly performed a subacute toxicity study of CDTHD. The results indicated that CDTHD at the maximum tolerance dose 86.4 g/kg⋅d did not show any toxicity compared with normal rats within 4 weeks. Rats administered once with ANIT exhibited liver injury with apparent cholestasis at 24 h, 36 h and 48 h. The serum levels of ALT, AST, TBIL, DBIL, ALP and γ-GT, serving as biomarkers of cholestasis, significantly increased in a time-dependent manner. Meanwhile, the bile flow rate decreased when ANIT was given. The results above were consistent with previous reports [21]. We found that CDTHD at 10.8, 21.6, 43.2, 86.4, 35.1 and 62.1 g/kg⋅d could attenuate biomarkers of cholestasis and increase the bile flow rate in dose-dependent degree. In addition, the abnormal histopathological changes induced by ANIT, associated with hepatocytes and cholangiocytes damage, were significantly attenuated by CDTHD at the doses ranging from 10.8 g/kg⋅d to 86.4 g/kg⋅d. Furthermore, administration of CDTHD at large dose of 86.4 g/kg⋅d and 62.1 g/kg⋅d almost entirely prevented the progression of ANIT-induced liver injury. Our results showed that CDTHD exerted an obvious therapeutic effect on ANIT-induced cholestasis in a dose-dependent manner. Particularly, CDTHD at large dose from 35.1 to 86.4 g/kg⋅d were potent to cholestasis.

Correspondence analysis provided a visualized impress of diverse data relationship. It was used to analyze the integrated efficacy and similarity of different doses CDTHD in this research. The result provided 3 aspects of information. ①CDTHD doses ranging from 43.2 g/kg⋅d to 86.4 g/kg⋅d exhibited integrated protective effect in a dose-dependent manner. However, in the range of 5.4-10.8 g/kg⋅d, CDTHD had no obvious curative effect. ② The Euclidean distance sequence was M-N ≈ A-N ≈ B-N > C-N > D-N ≈ F-N > E-N ≈ G-N and N-M > E-M ≈ G-M > D-M ≈ F-M > C-M > B-M > A-M in all time and the distance increased as time went on. The result suggested that large dose CDTHD increased more potent efficacy than low or normal dose. Meanwhile, at the same dosage of Chi Shao such as D and F, E and G demonstrated similar efficacy. It also indicated that Chi Shao with large dose in formula accounted for the main reason for potent efficacy, whereas 3 other herbs, Dan Shen, Gua Lou and Ge Gen, only contributed to certain degree. ③ The groups were divided into 5 clusters according to CA result. The E and G groups as well as D and F groups were similar to normal rats it indicated the potent effect at these doses. Meanwhile, A, B and M groups were likely to gather together, suggesting a slight effect or even no effect on cholestatic hepatitis. In addition, when we compared the effect of F or G-doses with C-dose, it demonstrated that the effect on cholestatic hepatitis increased along with the increase the doses of Chi Shao in CDTHD formula. This result implied that Chi Shao might serve as one of the crucial ingredient in the CDTHD. Secondly, when we compared the effect of F and G-doses with D and E-doses respectively, it demonstrated that there was no difference between D and F in pharmacological effect, and so was E and G. It illustrated that when the doses of other ingredients (Dan Shen, Gua Lou and Ge Gen) decreased while Chi Shao remained at a constant dose, the pharmacological effect showed no differences. This comparison might prove the relatively auxiliary role of other ingredients and highlight the important role of Chi Shao in CDTHD from the contrary side.

In order to get a deep insight into its possible mechanism, phytochemical analysis was performed to reveal the main active components of CDTHD. Seven representative compounds including Gallic acid, Tanshinol II_A_, Puerarin, Albiflorin, Paeoniflorin, Benzoic acid and Salvianolic acid B were found by UPLC. Among them, Paeoniflorin, a monoterpene glycoside, is one of the most primary bioactive components in CDTHD. In our previous study, it has been proved to demonstrate liver protective effect in rats due to its antioxidant effect [33]. Apart from the antioxidant effect in liver, Paeoniflorin has been consistently shown to attenuate amyloid-beta peptide-induced neurotoxicity by ameliorating oxidative stress [34]. It can also protect endothelial cells from hypoxic damage by enhancing nitric oxide production and can prevent oxidative damage in human umbilical vein endothelial cells [35]. Furthermore, Tanshinol II_A_, Salvianolic acid B and Puerarin serving as the main compounds in Dan Shen and Ge Gen respectively also exhibited the obvious antioxidant effect on liver disease [36-38]. Therefore, the mechanism of CDTHD in treating cholestatic hepatitis is possibly via its antioxidant efficacy.

In all, our study on dose-effect relationship of CDTHD provided a useful reference for the rational application of CDTHD in clinic and also supplied the research model of large dose application in TCM. This study just illustrated a partial scientific connotation of large dose CDTHD application. What do other herbs in the formula act in cholestatic hepatitis treatment? What is the mechanism of large dose CDTHD in treating cholestatic hepatitis? We can not offer an exact answer at present. Therefore, there is still some extension for further investigation in large dose of CDTHD.

## Conclusions

CDTHD is beneficial to liver protection in a dose-dependent manner. Large dose of CDTHD demonstrates potent efficacy and *Radix Paeoniae* Rubra in the formula exerts the main effect on treating ANIT-induced acute cholestatic hepatitis without toxicity.

## Abbreviations

CDTHD: Chi-Dan-Tui-Huang decoction
CAM: Complementary and alternative medicine
TCMs: Traditional Chinese Medicines
ANIT: Alpha-naphthylisothiocyanate
ALT: Alanine aminotransferase
AST: Aspartate aminotransferase
TBIL: Total bilirubin
DBIL: Direct bilirubin
ALP: Alkaline phosphatase
γ-GT: γ-glutamyl transpeptidase
